# Inter-eye Differences in Ocular Biometric Parameters of Concomitant Exotropia

**DOI:** 10.3389/fmed.2021.724122

**Published:** 2022-01-04

**Authors:** Weifen Gong, Haoyu Chen, Fan Yang, Shibin Lin, Chao Li, Geng Wang

**Affiliations:** Joint Shantou International Eye Center of Shantou University and The Chinese University of Hong Kong, Shantou, China

**Keywords:** concomitant exotropia, ocular biometric parameters, OA-2000, intereye differences, anisometropia

## Abstract

**Purpose:** To evaluate the ocular biometric parameters in patients with constant and intermittent exotropia by the measurement of swept-source optical coherence tomography (SS-OCT) optical biometer OA-2000 and comparing it with the normal control subjects.

**Design:** Cross-sectional case-control study.

**Participants:** Fifty-five constant and 24 intermittent patients with exotropia with central fixation and 77 orthotropic normal control participants aged 4–18 years old.

**Methods:** Non-contact and high-resolution optical biometric OA-2000 measurements were conducted under uniform ambient light conditions. The statistical analysis included intraclass correlation coefficient (ICC), Bland-Altman plot, and independent *t*-tests.

**Main Outcome Measures:** Spherical equivalent (SE), ocular biological parameters such as pupil diameter (PD), anterior chamber depth (ACD), lens thickness (LT), and axial lengths (AL). The absolute values of inter-eye differences for SE, PD, ACD, LT, and AL were recorded as AnisoSE, AnisoPD, AnisoACD, AnisoLT, and AnisoAL, respectively.

**Results:** AnisoSE (0.878 vs. 0.577, *P* = 0.019), AnisoAL (0.395 vs. 0.208, *P* = 0.001), AnisoACD (0.060 vs. 0.032, *P* < 0.001), AnisoLT (0.060 vs. 0.031, *P* = 0.002), and AnisoPD (0.557 vs. 0.340, *P* = 0.002) were significantly larger in concomitant patients with exotropia. The SE, AL, ACD, LT, and PD showed excellent binocular correlation with ICC values that ranged from 0.943 to 0.987 in control participants and from 0.767 to 0.955 in concomitant exotropia patients. Bland-Altman plots showed the wider range of agreement in patients with concomitant exotropia than the control participants (SE: 5.0288 vs. 3.3258; AL: 2.2146 vs. 1.3172; ACD:0.3243 vs. 0.1682; PD: 2.4691 vs. 1.9241; and LT:0.3723 vs 0.1858).

**Conclusion:** Patients with concomitant exotropia showed larger inter-eye differences in SE, ACD, LT, PD, and AL. Advice should be given to suspicious children to avoid or delay the development of concomitant exotropia.

## Introduction

Concomitant exotropia is a manifest divergent strabismus, defined as the deviating angle independent of gaze direction. It affects ~4% of the adult population (1) and could lead to the loss of binocularity and stereopsis. Besides the functional effects, patients with concomitant exotropia experience significant psychological stress, anxiety, and depression (2, 3). Concomitant exotropia often negatively impacts self-esteem, self-confidence, and interpersonal relationships (4, 5). Some patients assume adaptive techniques to hide exotropia, such as placing their hair over the deviating eye (2). Adults with strabismus often have reduced quality of life (6), lower education levels, and fewer career choices (7).

The cause of concomitant exotropia is still controversial. The reported risk factors included maternal smoking during pregnancy (8), premature birth, perinatal morbidity, genetic anomalies (9), family history, anisometropia (10, 11), and myopia (12). Some studies (13, 14) have reported the contributions of rectus extraocular muscles, and revealed the strong relationship between extraocular muscle's pulley position and refraction. Refractive errors, especially myopia and anisometropia, have a strong correlation with ocular biometric parameters, especially in axial length (15–18). Yet, the correlation between ocular biometric parameters and concomitant exotropia remains unclear, and few studies have evaluated the ocular biometric parameters in concomitant exotropia.

Ocular biometric measurements have been widely used in clinical practices such as the calculation of intraocular lens (IOL) power (19), candidate screening for refractive surgery, monitoring of the ametropic progression (20), estimation of the ocular mechanical properties (21), measurement of ocular anatomic changes on different accommodative stimuli (22, 23), or wearing contact lenses (24). As a novel non-contact and high-resolution optical biometric device, OA-2000 (Tomey, Nagoya, Japan) incorporates swept-source optical coherence tomography and a Placido-disc topographer, which could automatically find a measurable point and complete the scans quickly and accurately. It shows high repeatability and reproducibility, and an excellent agreement with other optical biometric devices, such as IOL Master700 (Carl Zeiss Meditec AG, Jena, Germany) and Lenstar-LS900 (Haag Streit AG, Koeniz, Switzerland) (18, 20, 25, 26). In this study, we aimed to evaluate the ocular biometric parameters in patients with constant and intermittent exotropia by OA-2000 and compare this with the normal control subjects to determine the contribution of ocular biometric parameters in concomitant exotropia.

## Methods

### Study Subjects

The study protocol conformed to the Declaration of Helsinki and was approved by the Human Medical Research Ethics Committee of Joint Shantou International Eye Center of Shantou University and by the Chinese University of Hong Kong. Informed consent was obtained from the legal representatives of all subjects after a full explanation of the purpose of the study had been fully accomplished. Patients with concomitant exotropia aged 4–18 years old were recruited between July and August 2019. All subjects underwent comprehensive ophthalmic examinations, including the measurements of visual acuity, intraocular pressure, cycloplegic refraction, anterior segment, and fundus examination, corneal light reflex tests, prism alternate cover test (PACT) for ocular alignment measurement, fixation behavior, and OA-2000 for ocular biometric parameter collection. A total of 79 patients, consisting of 55 constant and 24 intermittent concomitant exotropia with central fixation, were recruited. Among the 24 intermittent patients, 10 patients had reduced stereopsis tested by Titmus, 800 arc in six patients, and 400 arc in four patients. All other patients had lost stereopsis. A total of 77 control subjects were recruited with normal ocular motility, stereopsis, and binocular alignment. Subjects with any previous eye surgery, structural ocular anomalies, amblyopia of either eye, ptosis, cataract, and nystagmus were excluded.

### Ophthalmic Examinations

Visual acuity was measured using the Snellen chart. Cycloplegia was obtained by using compound tropicamide eye drops in children more than 6 years old, or atropine in 4-6-year-old subjects. Objective and subjective refraction was conducted, and refractive status was recorded as spherical equivalent (SE) [spherical power + (cylindrical power)/2]. Flat keratometry (K1), steep keratometry (K2), central corneal thickness (CCT), white to white distance (WTW), pupil diameter (PD), anterior chamber depth (ACD), lens thickness (LT), and axial lengths (AL) were measured with OA-2000 before the cycloplegia. Every subject had more than eight records taken at one time with a single OA-2000 measurement under uniform ambient light conditions. Only measurements with high accuracy (SD < 0.04 for keratometry, 0.02 for other parameters) and with level A were acceptable; otherwise, re-measurement was conducted. The absolute values of inter-eye differences for SE, PD, ACD, LT, and AL were recorded as AnisoSE, AnisoPD, AnisoACD, AnisoLT, and AnisoAL.

### Statistical Analysis

The within-group inter-eye differences were analyzed by the intraclass correlation coefficient (ICC) and Bland-Altman plot. ICCs were classified as excellent with values >0.90, good with values between 0.75 and 0.9, moderate with values between 0.5 and 0.75, and poor with values <0.5. An independent sample *t*-test was used to compare age, refractive status, ocular biometric measurements, and absolute values of inter-eye differences. Mann-Whitney *U*-test was used if equal variances were not assumed in Levene's Test. The *P* < 0.05 was considered as statistically significant.

## Results

A total of 156 study subjects, including 79 concomitant exotropia patients (mean age ± SD: 9.92 ± 3.32) and 77 control subjects (mean age ± SD: 8.97 ± 3.11), were recruited with 32 females and 47 males in patients with concomitant exotropia, and 36 females and 41 males in control subjects. AnisoSE (*t* 2.369, P.019) was significantly larger in concomitant exotropia patients with ICC values of 0.877 in patients with concomitant exotropia and 0.943 in control subjects (Tables 1, 2). No statistically significant difference was found in gender and age.

**Table 1:** Comparisons of the absolute values of inter-eye differences between concomitant exotropia and control participants.

	**XT mean (SD)**	**NCP mean (SD)**	***T*-value (*P*-value)**	**Mann-Whitney *U*-test**
AnisoSE (D)	0.878 (0.931)	0.577 (0.625)	2.369 (0.019^*^)	0.034
AnisoAL (mm)	0.395 (0.404)	0.208 (0.265)	3.423 (0.001^*^)	< 0.001
AnisoACD (mm)	0.060 (0.059)	0.032 (0.030)	3.782 (<0.001^*^)	0.001
AnisoPD (mm)	0.557 (0.476)	0.340 (0.357)	3.229 (0.002^*^)	0.001
AnisoLT (mm)	0.060 (0.073)	0.031 (0.035)	3.136 (0.002^*^)	0.003

*XT, concomitant exotropia; NCP, normal control participants; AnisoSE, absolute values of intereye differences for spherical equivalent; AnisoAL, absolute values of intereye differences for axial length; AnisoACD, absolute values of intereye differences for anterior chamber depth; AnisoPD, absolute values of intereye differences for pupil diameter; AnisoLT, absolute values of intereye differences for lens thickness*.

* *Results with equal variances not assumed and reanalyzed with Mann-Whitney U-test*.

**Table 2:** Correlation coefficients of the ocular biometric parameters between the two eyes within concomitant exotropia and control participants.

	**XT**			**NCP**		
	**ICC** **(*P*-value)**	**Pearson's correlation**	**Pearson's correlation *P***	**ICC** **(*P*-value)**	**Pearson's correlation**	**Pearson's correlation *P***
SE	0.877	0.876	<0.001	0.943	0.944	<0.001
AL	0.914	0.915	<0.001	0.971	0.972	<0.001
ACD	0.955	0.956	<0.001	0.987	0.987	<0.001
PD	0.767	0.826	<0.001	0.944	0.945	<0.001
LT	0.924	0.924	<0.001	0.972	0.974	<0.001

*XT, concomitant exotropia; NCP, normal control participants; ICC, intraclass correlation coefficient; SE, spherical equivalent; AL, axial length; ACD, anterior chamber depth; PD, pupil diameter; LT, lens thickness*.

### OA-2000 Findings

No significant difference of either the left or the right eye was found in K1, K2, CCT, WTW, PD, ACD, LT, and AL between concomitant exotropia and the control subjects. AnisoAL, AnisoACD, AnisoPD, and AnisoLT were statistically significant in concomitant exotropia patients with unassumed equal variances. Therefore, these parameters were analyzed with a non-parametric Mann-Whitney *U*-test, finding that concomitant exotropia patients showed larger AnisoAL, AnisoACD, AnisoPD, and AnisoLT (Table 1).

Table 2 demonstrated the correlation coefficients of the ocular biometric parameters between the two eyes within concomitant exotropia and control subjects. AL (ICC = 0.914, *r* = 0.915), ACD (ICC = 0.955, *r* = 0.956), PD (ICC = 0.767, *r* = 0.826), and LT (ICC = 0.924, *r* = 0.924) showed good to excellent binocular correlation in concomitant exotropia patients. AL (ICC = 0.971, *r* = 0.972), ACD (ICC = 0.987, *r* = 0.987), PD (ICC = 0.944, *r* = 0.945), and LT (ICC = 0.972, *r* = 0.974) showed excellent binocular correlation in control subjects.

### Bland-Altman Plots Findings

**Figure 1:**
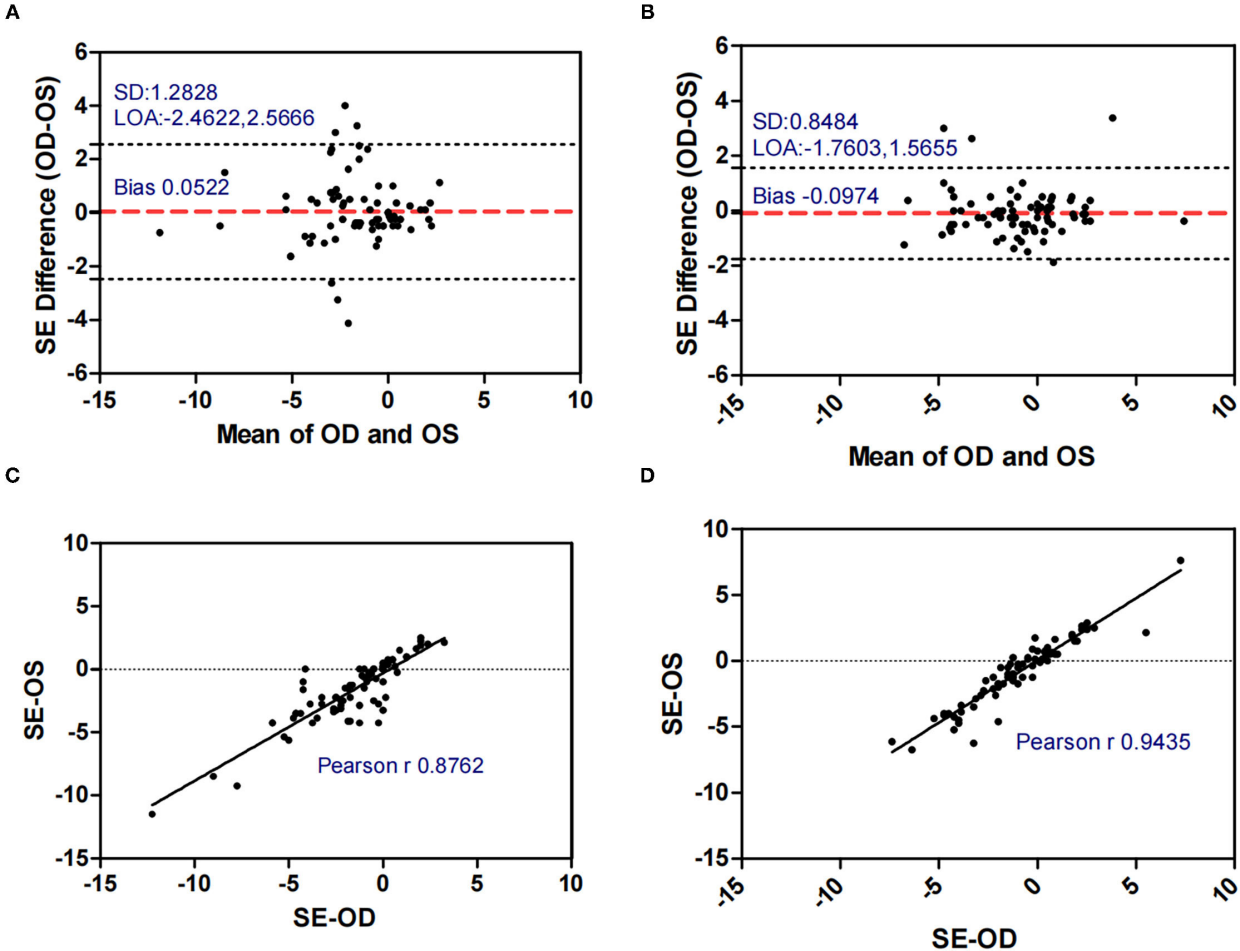
Bland-Altman plots **(A,B)** and scatter plots **(C,D)** of SE in concomitant exotropia **(A,C)** and normal control participants **(B,D)** of the left and right eyes. OS, left eye; OD, right eye; SE, spherical equivalent; LOA, 95% limits of agreement.

**Figure 2:**
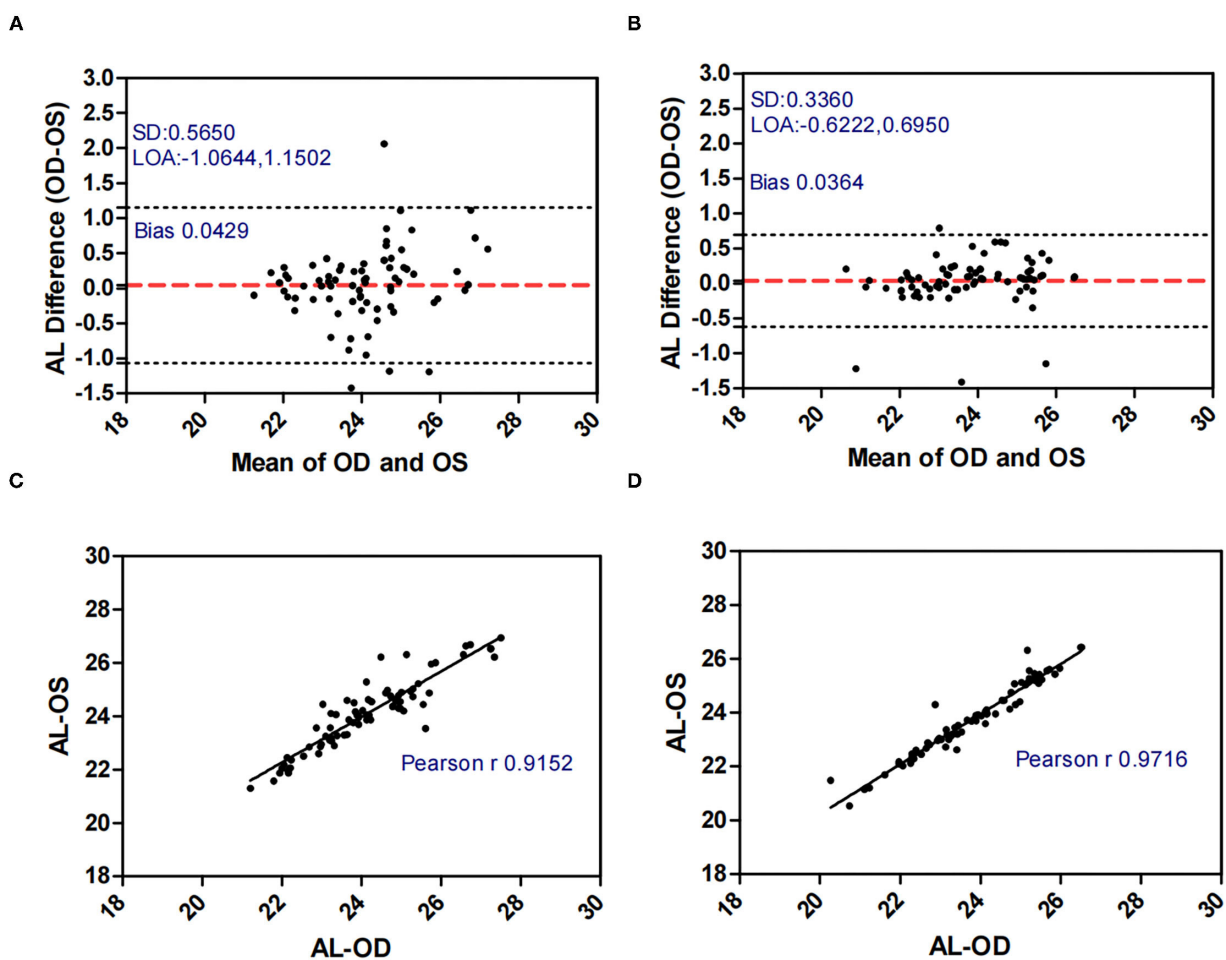
Bland-Altman plots **(A,B)** and scatter plots **(C,D)** of AL in concomitant exotropia **(A,C)** and normal control participants **(B,D)** of the left and right eyes. OS, left eye; OD, right eye; AL, axial length; LOA, 95% limits of agreement.

**Figure 3:**
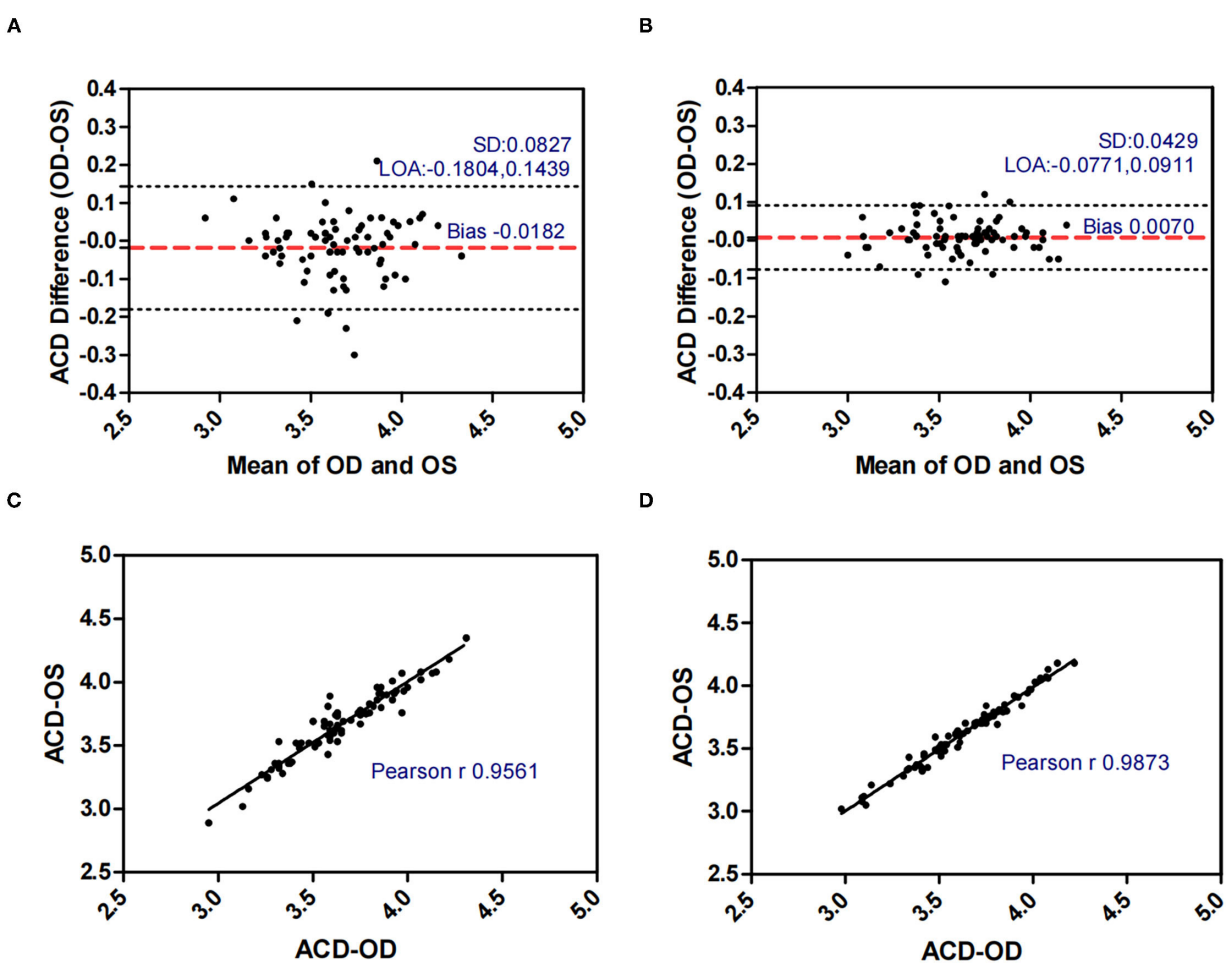
Bland-Altman plots **(A,B)** and scatter plots **(C,D)** of ACD in concomitant exotropia **(A,C)** and normal control participants **(B,D)** of the left and right eyes. OS, left eye; OD, right eye; ACD, anterior chamber depth; LOA, 95% limits of agreement.

**Figure 4:**
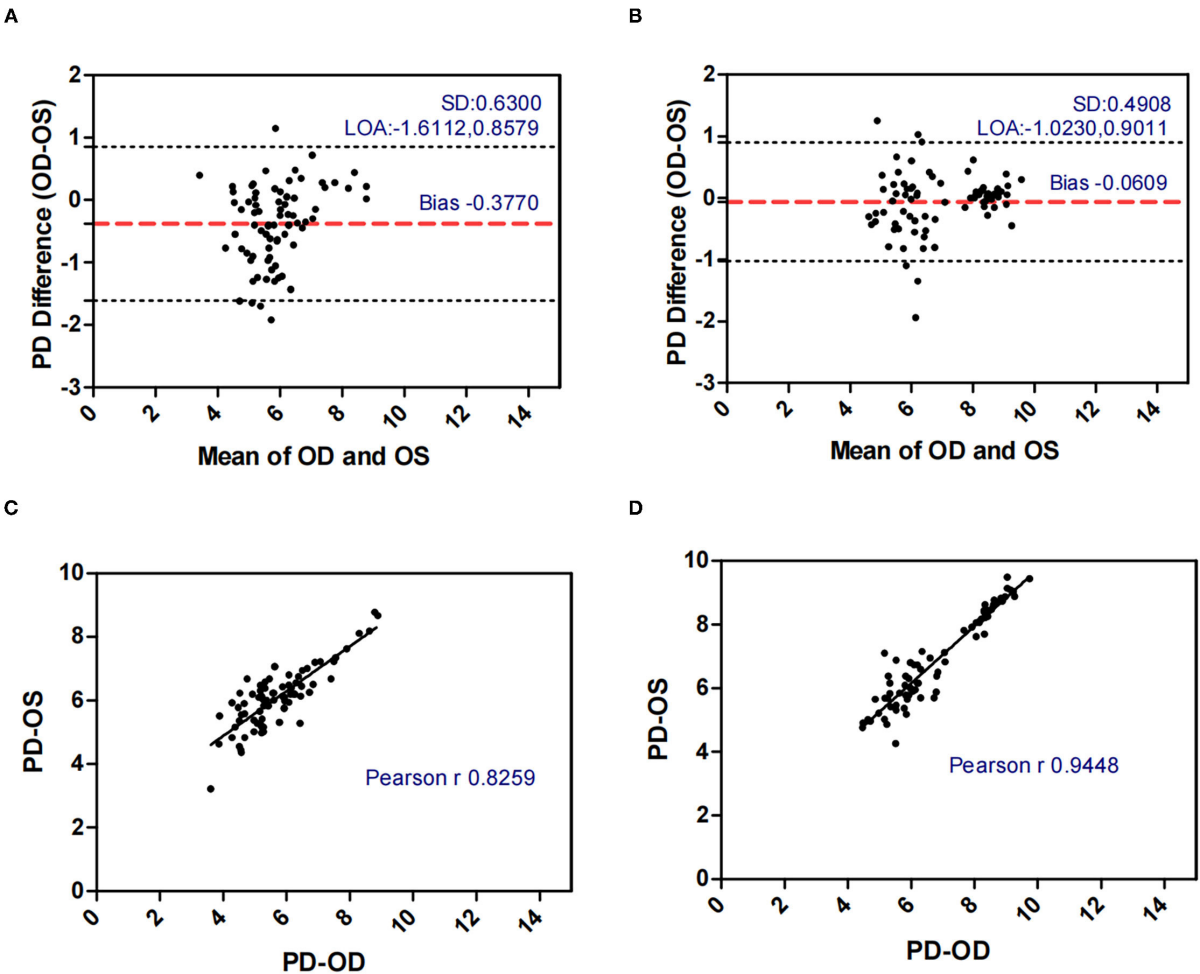
Bland-Altman plots **(A,B)** and scatter plots **(C,D)** of PD in concomitant exotropia **(A,C)** and normal control participants **(B,D)** of the left and right eyes. OS, left eye; OD, right eye; PD, pupil diameter; LOA, 95% limits of agreement.

**Figure 5:**
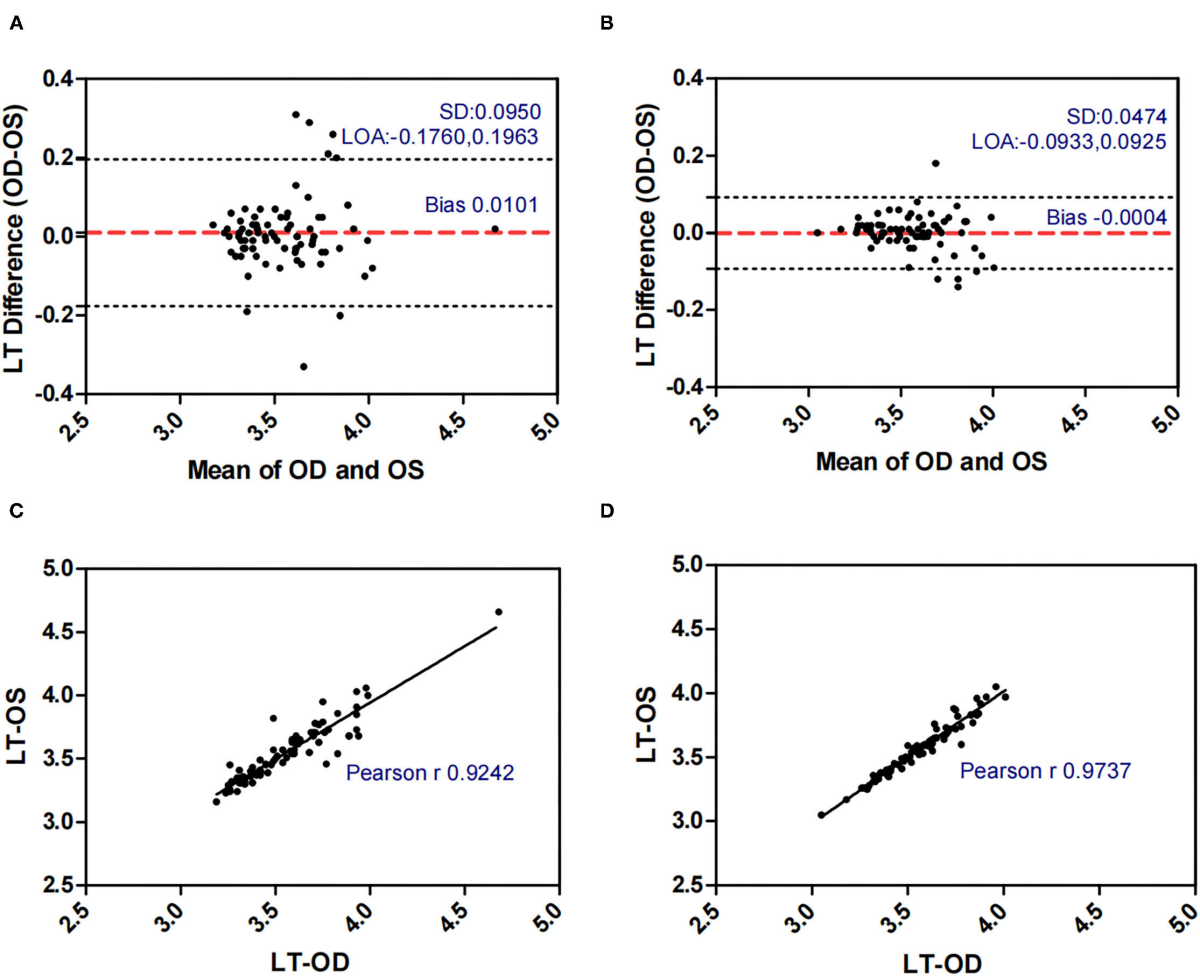
Bland-Altman plots **(A,B)** and scatter plots **(C,D)** of LT in concomitant exotropia **(A,C)** and normal control participants **(B,D)** of the left and right eyes. OS, left eye; OD, right eye; LT, lens thickness; LOA, 95% limits of agreement.

Figures 1–5 presented the Bland-Altman plots of the inter-eye differences in SE, AL, ACD, PD, and LT in concomitant exotropia (A,C) and also the normal control participants (B,D) of the left and right eyes. The ranges of agreement of the biometric parameters were listed in Table 3.

**Table 3:** Summary of the inter-ocular agreement of biometric parameters between concomitant exotropia and control participants.

	**XT**				**NCP**			
	**Mean difference**	**95% CI** **upper**	**95% CI** **lower**	**Range of agreement**	**Mean difference**	**95% CI** **upper**	**95% CI** **lower**	**Range of agreement**
SE (D)	0.0522 ± 1.2828	−2.4622	2.5666	5.0288	−0.0974 ± 0.8484	−1.7603	1.5655	3.3258
AL (mm)	0.0429 ± 0.5650	−1.0644	1.1502	2.2146	0.0364 ± 0.3360	−0.6222	0.6950	1.3172
ACD (mm)	−0.0182 ± 0.0827	−0.1804	0.1439	0.3243	0.0070 ± 0.0429	−0.0771	0.0911	0.1682
PD (mm)	−0.3770 ± 0.6300	−1.6112	0.8579	2.4691	−0.0609 ± 0.4908	−1.0230	0.9011	1.9241
LT (mm)	0.0101 ± 0.0950	−0.1760	0.1963	0.3723	−0.0004 ± 0.0474	−0.0933	0.0925	0.1858

*XT, concomitant exotropia; NCP, normal control participants; SE, spherical equivalent; AL, axial length; ACD, anterior chamber depth; PD, pupil diameter; LT, lens thickness*.

## Discussion

This study, for the first time, evaluated the ocular biometric features in patients with concomitant exotropia. In the present study, AnisoSE, AnisoAL, AnisoACD, AnisoPD, and AnisoLT were significantly larger in patients with concomitant exotropia than in control subjects. SE, AL, ACD, PD, and LT showed good to excellent binocular correlation with high ICC values ranging from 0.767 to 0.955 in concomitant exotropia patients and a range from 0.943 to 0.987 in control participants.

### Interpretation of the Results

Larger biometric inter-eye differences in patients with concomitant exotropia could be related to asymmetric binocular accommodative response. Previous studies reported the asymmetric binocular accommodative response with decreased accommodation in the deviating eye (27) and increased accommodative loads for binocular fusion (28) in patients with concomitant exotropia. The decreased accommodation reflected a primary sensory loss over the central retinal region as a result of prolonged, early, and abnormal visual experience that is associated with abnormal interaction between the eyes, as well as the presence of strabismus and/or anisometropia (29).

Anisometropia had been reported to be associated with concomitant exotropia (17, 30–32). On the other hand, early-onset concomitant exotropia could lead to anisometropia due to the disruption of the emmetropization process (11). In our study, anisometropia was significantly larger in concomitant exotropia patients (mean: 0.878) as compared to the control subjects (mean: 0.577). The SE in the control subjects showed excellent binocular correlation (ICC = 0.943, *r* = 0.944) and narrow range of agreement (Bias: −0.097, 95% limits of agreement: −1.760, 1.566), while the binocular correlation in concomitant exotropia patients was weaker (ICC = 0.877, *r* = 0.876) with a wider range of agreement (Bias: 0.052, 95% limits of agreement: −2.462, 2.567). Our study confirmed the association between anisometropia and concomitant exotropia.

Other biometric components such as AL, LT, and ACD are important in anisometropia (15), especially AL. AL as an indicator of myopic progression (33), had a strong correlation with anisometropia (15, 34). In our study, AnisoAL was significantly larger (0.395 vs 0.208) with lower ICC (0.914 vs 0.971) in patients with concomitant exotropia.

LT and ACD, as the important factors in anisometropia, contribute to refractive error. During accommodation, the ACD decreases and the LT increases (35). Inter-eye differences in LT and ACD revealed the asymmetric accommodative response between two eyes. In our study, AnisoLT and AnisoACD were significantly larger (0.060 vs 0.031) in concomitant exotropia patients, indicating more inter-eye differences in accommodation. The control subjects had higher intra-class correlation (ICC = 0.972 in LT, ICC = 0.987 in ACD) and narrower range of agreement than patients with concomitant exotropia (ICC = 0.924 in LT, ICC = 0.955 in ACD).

Pupil diameter (PD) decreases with increasing age, retinal illumination, and near response. In our study, OA-2000 measurements were conducted under uniform ambient light conditions. The relationship between PD and concomitant exotropia is less frequently investigated in previous studies. In our study, concomitant exotropia patients showed larger AnisoPD (0.557 vs 0.340). The PD in the control subjects had an excellent binocular correlation (ICC = 0.944, *r* = 0.945) and had a narrow range of agreement (Bias: −0.061, 95% limits of agreement: −0.023, 0.901). Yet, the binocular correlation in patients with concomitant exotropia was weaker (ICC = 0.767, *r* = 0.826), with a wider range of agreement (Bias: −0.377, 95% limits of agreement: −1.611, 0.858). Notably, Pearson's correlation was higher than ICC in patients with concomitant exotropia due to the difference in statistical analyses. The data were centered and scaled using a pooled mean and a standard deviation in ICC, but each variable was centered and scaled by its own mean and SD in the Pearson's correlation (36).

In conclusion, larger biometric inter-eye differences may be caused by concomitant exotropia itself, asymmetric binocular accommodation, anisometropia, or the interaction of these factors.

## Strengths

Unlike other technologies such as MRI, which has a superior soft-tissue contrast, evaluates both anatomic and physiologic parameters simultaneously, and is particularly useful in rectus extraocular muscle evaluation (14), the optical biometry, such as OA 2000, has been proven to be more accurate and safer for ocular biometric measurements (37, 38). Only one measurement was taken for each subject, which was time-saving due to the high repeatability and reproducibility. By OA 2000 measurements, we revealed larger AnisoSE, AnisoAL, AnisoACD, AnisoLT, and AnisoPD in patients with concomitant exotropia. Subjects with larger AnisoSE, AnisoAL, AnisoACD, AnisoLT, and AnisoPD were prone to develop concomitant exotropia. Advice should be given to the suspicious subjects to avoid or delay the development of concomitant exotropia.

## Limitations

There were several limitations in this study. First, this was a cross-sectional study. Second, the study subjects were not randomly selected. Third, among all parameters measured by OA 2000, the measurements of PD should be adjusted manually by two technicians. Fourth, every subject with central fixation in our study had only one measurement, although more than 8 results were recorded. The measurement biases could not be avoided, even though OA-2000 shows high repeatability and reproducibility.

In summary, our study initially evaluated the ocular biometric features in patients with concomitant exotropia and found that AnisoSE, AnisoPD, AnisoAL, AnisoACD, and AnisoLT could contribute to concomitant exotropia. Our results will contribute to the etiology and management of concomitant exotropia.

## Data Availability Statement

The original contributions presented in the study are included in the article/supplementary material, further inquiries can be directed to the corresponding author/s.

## Ethics Statement

The studies involving human participants were reviewed and approved by the Human Medical Research Ethics Committee of Joint Shantou International Eye Center of Shantou University and the Chinese University of Hong Kong. Written informed consent to participate in this study was provided by the participants' legal guardian/next of kin.

## Author Contributions

WG: study design, data collection, data analysis, and manuscript preparation. HC: data analysis and manuscript preparation. FY, CL, and SL: data collection. GW: study design and manuscript preparation. All authors contributed to the article and approved the submitted version.

## Funding

This study was supported by Shantou Science and Technology Project (Grant No. 190629115260318), Guangdong Medical Research Foundation (CN) (Grant No. A2016514), Shantou Municipal Science and Technology Project (Grant No. 190917155269927), and 2020 Li Ka Shing Foundation Cross-Disciplinary Research Grant (Project Number: 2020LKSFG06B).

## Conflict of Interest

The authors declare that the research was conducted in the absence of any commercial or financial relationships that could be construed as a potential conflict of interest.

## Publisher's Note

All claims expressed in this article are solely those of the authors and do not necessarily represent those of their affiliated organizations, or those of the publisher, the editors and the reviewers. Any product that may be evaluated in this article, or claim that may be made by its manufacturer, is not guaranteed or endorsed by the publisher.
